# Beyond amyloid: altered gene function in neurodegenerative diseases

**DOI:** 10.18632/aging.204717

**Published:** 2023-09-20

**Authors:** Metin Yesiltepe, Tao Yin, Luciano D’Adamio

**Affiliations:** 1 Department of Pharmacology, Physiology and Neuroscience New Jersey Medical School, Brain Health Institute, Jacqueline Krieger Klein Center in Alzheimer’s Disease and Neurodegeneration Research, Rutgers, The State University of New Jersey, Newark, NJ 07103, USA

**Keywords:** APP, Aβ, Alzheimer Disease, Itm2b, synaptic plasticity

The amyloid hypothesis is the dominant theory in Alzheimer’s Disease (AD), proposing that Aβ oligomers and plaques cause pathogenesis. However, the presence of amyloid in healthy individuals and the lack of amyloid pathology in AD-like cognitive decline suggests other mechanisms. Poor results from clinical trials of amyloid-reducing drugs confirm skepticism toward the hypothesis.

Our lab has focused on the function/dysfunction hypothesis in AD for nearly three decades. This hypothesis proposes that AD-linked mutations change critical functions of genes expressed in CNS, ultimately leading to disease pathogenesis. *APP* encodes Aβ precursor protein (APP), which is one of the genes we have studied because it is the Aβ precursor and pathogenic mutations in APP result in early-onset Familial AD. We have explored the synaptic functions of APP, with an emphasis on its role in synaptic vesicles (SV), after discovering that APP is present in SV, is highly enriched in presynaptic termini, and that it establishes an interactomic network with SV protein. We have identified two distinct domains of APP - the cytosolic (JCasp) and intravesicular (ISVAID) domains - that interact with SV proteins and modulate exocytosis. Specifically, interactions between SV proteins and the JCasp domain increase the release probability (*Pr*) of glutamatergic synapses [1], while interactions between SV proteins and the ISVAID domain decrease *Pr* [2].

**Figure 1:**
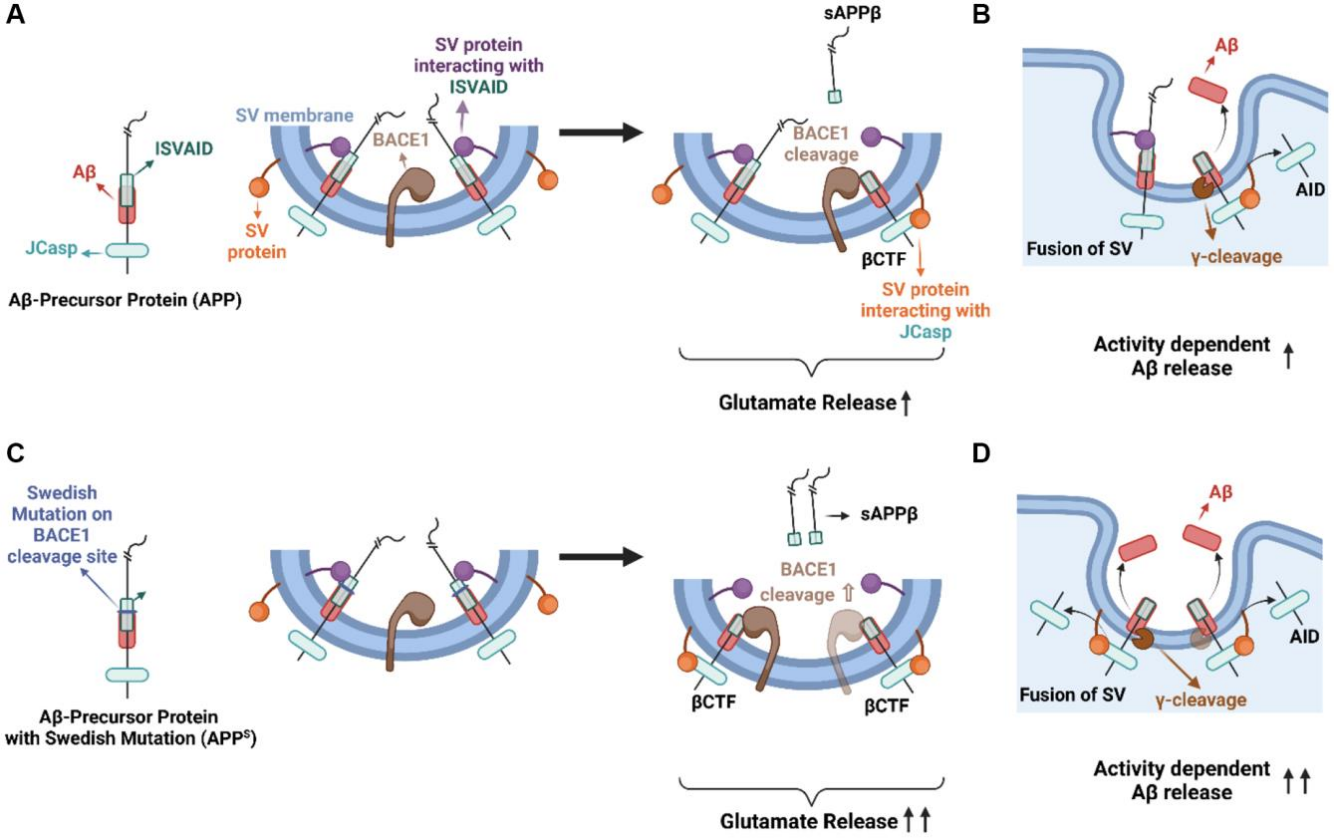
**Summary of BAD-Glu Phenomenon.** Exploring the role of APP and the Swedish mutation in BAD-Glu mechanisms and glutamate release. (**A**) APP interacts with SV proteins via intraluminal (ISVAID) and cytosolic (JCasp) domains, with opposite effects on glutamate release. BACE1 cleavage of APP can disrupt ISVAID-SV protein interaction and trigger facilitation of JCasp-SV protein interaction, which tune-up SV exocytosis. (**B**) During fusion of synaptic vesicles to the presynaptic membrane, γ-secretase cleaves βCTF and releases Aβ in the synaptic area. (**C**) Swedish mutation on BACE1 cleavage site cause an increased BACE1 cleavage and this may promote glutamate release. (**D**) During SV fusion, the increase in βCTF due to increased BACE1 cleavage may lead to an increase in Aβ release into the synaptic area. Created with https://biorender.com/.

BACE1 is one of the two proteases responsible for generating Aβ from APP and its cleavage site is located within the ISVAID segment. In the acidic environment of the SV, BACE1 activity is favored, leading to the cleavage of APP within the ISVAID region. This generates soluble fragments (sAPPβ) and membrane-bound carboxyl terminus (βCTF), the precursor of Aβ, and disrupts the intravesicular APP- SV interactions (Figure 1). As a result, this disruption leads to an increase in the release probability (*Pr*) of glutamatergic synapses [2]. These findings led us to propose the BACE1 on APP-Dependent Glutamate release model (BAD-Glu), which suggests that cleavage of APP by BACE1 within SV promotes glutamate release through the facilitation of βCTF-SV cytosolic interactions [3]. During exocytosis, when SV fuses with the presynaptic membrane, βCTF is processed to Aβ by γ-secretase processing. The fact that glutamatergic transmission increases Aβ production supports this model [4].

Is BAD-Glu altered by pathogenic mutations in APP? The pathogenic APP Swedish mutation enhances the APP cleavage by BACE1, increasing the production of sAPPβ and βCTF. This results in elevated Aβ peptide production as a secondary effect, which is used to support the amyloid cascade hypothesis. To test the role of β-secretase cleavage of APP in excitatory neurotransmission, we generated knock-in rats with the Swedish APP mutations (*App^s^* rats). These rats showed increased BACE1 processing of APP, Aβ production, and glutamate release, which is consistent with the BAD-Glu model [3].

Our recent study on 11-14-month-old *App^s^* rats revealed sex-specific differences caused by the Swedish mutation [5]. *App^s^* females performed significantly worse than same-sex controls in a spatial discrimination and flexibility task, while males *App^s^* did not show any significant deficits. Both male and female *App^s^* rats exhibited a similar significant increase in Aβ production without amyloid plaque pathology or significant neuroinflammation. The Swedish mutation resulted in significant impairment in long-term potentiation (LTP) in female *App^s^* rats compared to control females, but not in male *App^s^* rats, supporting a correlation between the Swedish mutation and synaptic plasticity and memory rather than Aβ levels and amyloidosis.

These two pathogenic hypotheses for AD have important but distinctive therapeutic implications. The amyloid hypothesis suggests reducing Aβ production and/or deposition to prevent or delay AD caused by the Swedish mutation. Inhibiting BACE1 and/or γ-cleavage of APP or targeting Aβ levels should be beneficial. However, the BAD-Glu hypothesis suggests reducing BACE1 processing of APP as a potential therapeutic strategy, while inhibiting γ-cleavage of βCTF could be harmful by stabilizing βCTF and potentially increasing BAD-Glu. Targeting Aβ levels would not be effective.

Do other genes involved in dementia’s pathogenesis follow the function/dysfunction hypothesis? *ITM2B/BRI2* mutations cause several autosomal dominant neurodegenerative diseases, including Familial Danish (FDD) and British (FBD) dementia that share similarities with AD, such as amyloid plaques and neurofibrillary tangles. All pathogenic ITM2B/BRI2 mutations lead to amyloidogenic peptide accumulation, which is believed to cause neuronal damage and dementia similar to the amyloid hypothesis. However, our research on *Itm2b-KO* mice shows that BRI2 plays a critical role in synaptic transmission and plasticity in glutamatergic neurons, which are also impaired in FDD and FBD knock-in rodent models [6–8]. Moreover, mutant forms of precursor BRI2 protein are unstable, leading to a decrease in overall protein levels, suggesting a loss of BRI2 protein function may contribute to the development of these diseases as well.

In conclusion, while the world’s attention is still fixed on the amyloid hypothesis as the main culprit behind neurodegenerative disorders, the evidence presented above warrants a pause for reflection. It suggests that changes to the normal brain function of genes related to dementia, whether due to genetic mutations or environmental/epigenetic factors, could be a significant contributor to the pathogenesis of these disorders.
